# Clinical Benefit of Mepolizumab in Eosinophilic Granulomatosis With Polyangiitis for Patients With and Without a Vasculitic Phenotype

**DOI:** 10.1002/acr2.11571

**Published:** 2023-06-13

**Authors:** Benjamin Terrier, David R.W. Jayne, Bernhard Hellmich, Jane H. Bentley, Jonathan Steinfeld, Steven W. Yancey, Namhee Kwon, Praveen Akuthota, Paneez Khoury, Lee Baylis, Michael E. Wechsler, the EGPA mepolizumab study team

**Affiliations:** ^1^ Hôpital Cochin Paris France; ^2^ University of Cambridge Cambridge UK; ^3^ Klinik für Innere Medizin, Rheumatologie & Immunologie, Medius Kliniken, Universität Tübingen Kirchheim‐Teck Germany; ^4^ Clinical Statistics GSK, Brentford, Middlesex UK; ^5^ Clinical Sciences Respiratory, GSK Philadelphia Pennsylvania; ^6^ Respiratory Therapeutic Area GSK, Research Triangle Park North Carolina; ^7^ Clinical Sciences Respiratory, GSK, Brentford Middlesex UK; ^8^ University of California San Diego; ^9^ Human Eosinophil Section, Laboratory of Parasitic Diseases, National Institute of Allergy and Infectious Diseases National Institutes of Health Bethesda Maryland; ^10^ Global Medical Affairs GSK, Durham North Carolina; ^11^ National Jewish Health Denver Colorado

## Abstract

**Objective:**

To evaluate mepolizumab's efficacy in eosinophilic granulomatosis with polyangiitis (EGPA) with and without a vasculitic phenotype.

**Methods:**

The MIRRA study (NCT02020889/GSK ID: 115921) included adults with relapsing/refractory EGPA and 4 or more weeks of stable oral glucocorticoids (OG). Patients received mepolizumab (300 mg subcutaneously every 4 weeks) or placebo, plus standard of care for 52 weeks. This post hoc analysis assessed EGPA vasculitic phenotype using antineutrophil cytoplasmic antibody (ANCA) history, baseline Birmingham Vasculitis Activity Score (BVAS), and Vasculitis Damage Index (VDI) score. Coprimary endpoints included accrued remission over 52 weeks and proportion in remission at Week 36 and Week 48. Remission was defined as a BVAS equal to 0 and an OG dose of 4 or more mg/day of a prednisone equivalent. Types of relapses (vasculitis, asthma, and sino‐nasal) and EGPA vasculitic characteristics (by study remission status) were also assessed.

**Results:**

A total of 136 patients were included (n = 68, mepolizumab and placebo). Irrespective of history of ANCA positivity status, baseline BVAS, or baseline VDI, the accrued remission duration and the proportion of patients in remission at Weeks 36 and 48 were greater with mepolizumab compared with placebo. With mepolizumab, remission at both Week 36 and Week 48 was achieved by 54% of patients with and 27% of patients without a history of ANCA positivity compared with 0% and 4%, respectively (placebo); 45% of patients with a BVAS of 0 and 22% of patients with BVAS of greater than 0 compared with 5% and 2%, respectively (placebo); and 29% of patients with a VDI score of less than 5 and 37% of patients with a VDI score of 5 or more compared with 6% and 0%, respectively (placebo). Mepolizumab reduced all types of relapses as compared with placebo. Baseline vasculitic characteristics (neuropathy, glomerulonephritis, alveolar hemorrhage, palpable purpura, and ANCA positivity) were generally similar among patients with and without remission.

**Conclusion:**

Mepolizumab is associated with clinical benefits for patients with and without a vasculitic EGPA phenotype.

## INTRODUCTION

Eosinophilic granulomatosis with polyangiitis (EGPA) is a rare systemic inflammatory disorder associated with blood and tissue eosinophilia, asthma, and necrotizing vasculitis and granulomatous inflammation (1, 2). These characteristics often contribute to organ injury, impairment, and life‐threatening conditions (3, 4). EGPA can be divided into predominantly vasculitic or eosinophilic phenotypes based on clusters of often overlapping clinical features (5, 6). Common features of the vasculitic phenotype include the presence of palpable purpura, peripheral neuropathy, and glomerulonephritis (6). In contrast, the eosinophilic phenotype is more frequently characterized by lung infiltrates and cardiomyopathy and is often associated with elevated blood and tissue eosinophil counts (5, 6). As some vasculitis‐associated manifestations such as glomerulonephritis or palpable purpura occur more frequently in patients with a positive antineutrophil cytoplasmic antibody (ANCA) status, the vasculitic and eosinophilic phenotypes can be often inferred by ANCA status (5, 6). However, typically, only 30% to 40% of patients with EGPA have ANCA positivity (6). Of those with ANCA positivity, the majority (≥90%) have ANCA specific for myeloperoxidase (MPO), and MPO‐ANCA‐positive EGPA may have a genetic contribution distinct from ANCA‐negative EGPA (6, 7). Moreover, only a small minority of patients have proteinase 3 (PR3)‐positive ANCA (6, 8).

The standard treatment for EGPA consists of combination treatment with systemic glucocorticoids, immunosuppressants, cytotoxic therapy, and biologic therapy (9, 10, 11). However, immunosuppressant, glucocorticoid, and cytotoxic therapies are associated with considerable toxicity, and relapses are common despite continuous therapy (9, 12, 13, 14, 15, 16, 17, 18). In particular, side effects of glucocorticoid treatment include osteoporosis, fractures, infections, type 2 diabetes, and cardiovascular events, highlighting the need for glucocorticoid‐sparing therapy (13, 19).

Mepolizumab is a humanized monoclonal antibody that binds to and inactivates interleukin‐5 (IL‐5), leading to the depletion of eosinophils within the blood and tissues (20, 21). Mepolizumab is approved for the treatment of severe eosinophilic asthma, EGPA, hypereosinophilic syndrome, and chronic rhinosinusitis with nasal polyps across multiple regions worldwide (22, 23, 24). Because eosinophils can contribute to manifestations of both EGPA phenotypes, eosinophil‐targeting biologic therapy may be beneficial for patients with vasculitic or eosinophilic EGPA (6, 25). IL‐5 is a key cytokine responsible for the proliferation, maturation, activation, recruitment, and survival of eosinophils and is implicated in EGPA pathogenesis (26, 27, 28). In the Phase III MIRRA study, patients with relapsing or refractory EGPA receiving mepolizumab had a greater duration of disease remission and reduced use of oral glucocorticoids (OG) versus placebo (29). However, although the MIRRA study enrolled both patients with vasculitic and/or eosinophilic phenotypes of EGPA (29), the primary analysis was not designed to investigate the clinical benefits of mepolizumab in specific EGPA phenotypes.

The objective of this post hoc analysis was to evaluate the clinical benefits of mepolizumab by examining disease control and OG use in patients with and without a vasculitic EGPA phenotype, using data from the MIRRA study.

## PATIENTS AND METHODS

### Study design and patients

The design and eligibility criteria of the MIRRA study have been published previously (29). Briefly, MIRRA (GSK ID: 115921; NCT02020889) was a randomized, double‐blind, Phase III multicenter trial in which patients randomly received (1:1) mepolizumab (300 mg subcutaneously every 4 weeks) or placebo for 52 weeks, in addition to standard of care including OG with or without immunosuppressive therapy. From 4 weeks after baseline, a patient's OG dose could be reduced using a recommended schedule at the investigator's discretion. Patients using immunosuppressive therapy were required to maintain a stable dose throughout the trial.

Key patient eligibility criteria included being 18 years of age or older, having an EGPA diagnosis for 6 months or longer based on the presence of asthma and eosinophilia (>1000 cells/μL and/or >10% of leucocytes), and having two or more of the following additional features of EGPA: i) biopsy showing histopathological evidence of eosinophilic vasculitis, or perivascular eosinophilic infiltration, or eosinophil‐rich granulomatous inflammation; ii) neuropathy; iii) nonfixed pulmonary infiltrates; iv) sino‐nasal abnormality; v) cardiomyopathy; vi) glomerulonephritis; vii) alveolar hemorrhage; viii) palpable purpura; or ix) ANCA (MPO or PR3) positivity. Patients were also required to have a history of relapsing or refractory disease and be receiving a stable dose (≥7.5 to 50 mg/day) of OG for 4 weeks or longer before baseline. Relapsing disease was defined by one or more confirmed EGPA relapses (requiring an OG dose increase, initiation or increased dose of immunosuppressant therapy or intravenous immunoglobulin, or hospitalization) that occurred within the previous 2 years and that occurred 12 weeks or longer prior to screening while receiving 7.5 mg/day or more of a prednisone equivalent. Refractory disease was defined according to European League Against Rheumatism criteria (19) as failure to attain remission (Birmingham Vasculitis Activity Score [BVAS] equal to 0 and OG dose 7.5 mg/day or less of a prednisone equivalent) within the last 6 months following induction treatment with a standard regimen administered for 3 months or longer or within the 6 months prior to screening or as the recurrence of EGPA symptoms while tapering OG dose (≥7.5 mg/day of a prednisone equivalent). Patients were excluded if they had granulomatosis with polyangiitis or microscopic polyangiitis at screening, or organ‐ or life‐threatening EGPA in the 3 months before screening.

The trial was conducted in accordance with the ethical principles of the Declaration of Helsinki, the International Conference on Harmonisation Good Clinical Practice guidelines, and the applicable country‐specific regulatory requirements, and all the patients provided written informed consent.

### Identification of patients with and without a vasculitic EGPA phenotype

Patients were classified as likely to have a vasculitic phenotype based on three separate markers: 1) history of ANCA positivity, 2) baseline BVAS, or 3) baseline Vasculitis Damage Index (VDI) score. A baseline, or previous, positive test for MPO/PR3‐ANCA was used to define patients more likely to have a vasculitic EGPA phenotype (6, 7), whereas no history of ANCA positivity identified those less likely to have a vasculitic EGPA phenotype. Likelihood of vasculitic EGPA was also defined by a baseline BVAS of greater than zero (30) (vs. BVAS = 0 for patients less likely to have vasculitic EGPA). Finally, the likelihood of vasculitic EGPA was defined by a baseline VDI score of 5 or greater (vs. <5 for patients less likely to have vasculitic EGPA) as VDI scores of 5 or greater indicate more severe vasculitic disease. (31)

### Endpoints and assessments

The coprimary endpoints of the MIRRA study were the total accrued duration of remission and proportion of patients in remission at both Weeks 36 and 48. Remission was defined as a BVAS (version 3) of 0 (scale: 0 to 63, with higher scores indicating greater disease activity) (32) and an OG dose of 4.0 mg/day or less of a prednisone equivalent. Accrued duration of remission was categorized in weeks (0, >0 to <12, 12 to <24, 24 to <36, and ≥36 weeks).

Endpoints included in this post hoc analysis were as follows: assessment of differences in patient characteristics (focusing on vasculitic components of EGPA diagnostic disease characteristics [neuropathy, glomerulonephritis, alveolar hemorrhage, palpable purpura, and ANCA‐positive status]) by patient remission status (achieved/did not achieve remission at any time and accrued remission ≥36 weeks); assessment of mepolizumab versus placebo treatment effect on the coprimary endpoints in subgroups by ANCA status (current or previous positive test for MPO/PR3‐ANCA or no history of a positive MPO/PR3‐ANCA test at baseline). Prespecified analyses included baseline BVAS (score = 0 or >0) and VDI scores (<5 or ≥5); the proportion of patients with a relapse and number of relapse events (relapses were classified according to categories of vasculitis only, asthma only, sino‐nasal only, or combinations of categories [vasculitis/asthma, vasculitis/sino‐nasal, asthma/sino‐nasal, vasculitis/asthma/sino‐nasal], all relapses (included relapses without another category defined and respective combinations of categories), and categories with or without another relapse category defined; cumulative incidence of relapse over time for all relapses and according to type of relapse (asthma only, vasculitis only, sino‐nasal only); and total accrued duration of BVAS equal to 0 over 52 weeks and proportion of patients achieving a BVAS equal to 0 at both Weeks 36 and 48.

Although not specifically developed for EGPA, BVAS is validated for the assessment of 66 active vasculitis manifestations across nine organ systems (32). The VDI measures features of vasculitis due to persistent damage across 11 organ systems in patients with or without current disease activity (31).

For relapse categorization, a vasculitis relapse was defined as a BVAS of more than 0, an asthma relapse was defined as active asthma symptoms and/or signs with a corresponding worsening in Asthma Control Questionnaire (ACQ)‐6 score (compared with the most recent previous score), and sino‐nasal relapse was defined as active nasal and/or sinus disease with corresponding worsening in one or more of the sino‐nasal symptom questions (compared with the most recent previous assessment).

### Statistical analysis

Accrued duration of remission was categorized in weeks (0, >0 to <12, 12 to <24, 24 to <36 and ≥36) and analyzed using a proportional odds model with covariates of treatment, baseline prednisone daily dose, and baseline BVAS (excluding baseline BVAS analysis) and region (excluding analysis by historical ANCA status). The proportion of patients in remission at both Weeks 36 and 48 was analyzed using a logistic regression model with covariates of treatment, baseline prednisone daily dose, baseline BVAS (excluding baseline BVAS analysis), and region. Exploratory analyses of the interaction between baseline BVAS or baseline VDI score and mepolizumab treatment (vs. placebo) were performed, with baseline BVAS as a continuous and categorical variable (score = 0 or >0) and VDI score as a categorical (score <5 or ≥5) variable.

## RESULTS

### Patient characteristics by remission status

Of the 136 patients randomized to treatment (68 to mepolizumab and 68 to placebo), 49 achieved remission at any point during the 52‐week treatment period (36 mepolizumab, 13 placebo), and 11 patients accrued remission for 36 weeks or longer (9 mepolizumab, 2 placebo).

A similar proportion of patients (mepolizumab treated and placebo treated) who achieved and did not achieve remission had vasculitic manifestations of EGPA, as indicated by similar proportions of patients in each group who had a history or presence of neuropathy (39% and 43%), glomerulonephritis (0% and 1%), alveolar hemorrhage (4% and 2%), palpable purpura (12% and 13%) and a history of being ANCA positive (24% and 16%; Table 1). Of the seven patients in the overall population who were ANCA positive at screening, six were MPO‐ANCA positive, and one was PR3‐ANCA positive. For patients who did and did not accrue 36 weeks of remission or longer, results were generally similar, except that a higher proportion of patients who accrued 36 weeks of remission or longer had a history or presence of neuropathy compared with those who did not (64% and 39%; Table 1).

**Table 1:** Patient characteristics by remission status

	Achieved remission at any time		Accrued ≥36 wk remission	
	Yes	No	Yes	No
	(n = 49)	(n = 87)	(n = 11)	(n = 125)
Age, y, mean (SD)	47.7 (14.62)	48.9 (12.64)	51.6 (11.64)	48.2 (13.49)
Female, n (%)	28 (57)	52 (60)	7 (64)	73 (58)
EGPA disease duration (y), mean (SD)	5.9 (4.27)	5.4 (4.83)	4.8 (3.16)	5.6 (4.74)
Patients with a history or presence of EGPA diagnostic features, n (%)				
Asthma with eosinophilia	49 (100)	87 (100)	11 (100)	125 (100)
Biopsy^a^	18 (37)	38 (44)	3 (27)	53 (42)
Neuropathy, mono or poly^b^ ^,^ ^c^	19 (39)	37 (43)	7 (64)	49 (39)
Pulmonary infiltrates, nonfixed	32 (65)	66 (76)	8 (73)	90 (72)
Sino‐nasal abnormality	46 (94)	82 (94)	10 (91)	118 (94)
Cardiomyopathy^d^	6 (12)	14 (16)	1 (9)	19 (15)
Glomerulonephritis^d^, ^e^	0	1 (1)	0	1 (<1)
Alveolar hemorrhage^e^, ^f^	2 (4)	2 (2)	1 (9)	3 (2)
Palpable purpura^b^	6 (12)	11 (13)	2 (18)	15 (12)
ANCA positive (MPO or PR3)^b^	12 (24)	14 (16)	4 (36)	22 (18)
History of relapsing or refractory disease, n (%)				
≥1 relapse in prior 2 y	36 (73)	64 (74)	10 (91)	90 (72)
Refractory disease	26 (53)	48 (55)	2 (18)	72 (58)
Failed induction treatment	1 (2)	5 (6)	0	6 (5)
Recurrence of EGPA symptoms while tapering oral glucocorticoid, n (%)	25 (51)	43 (49)	2 (18)	66 (53)

Abbreviations: ANCA, antineutrophil cytoplasmic antibody; EGPA, eosinophilic granulomatosis with polyangiitis; MPO, myeloperoxidase; MRI, magnetic resonance imaging; PR3, proteinase 3; SD, standard deviation.

^a^ A biopsy showing histopathological evidence of eosinophilic vasculitis, or perivascular eosinophilic infiltration, or eosinophil‐rich granulomatous inflammation.

^b^ Characteristics considered to be indicative of vasculitic EGPA.

^c^ Motor deficit or nerve conduction abnormality.

^d^ Established by echocardiography or MRI.

^e^ Hematuria, red cell casts, proteinuria.

^f^ Established by bronchoalveolar lavage.

### Remission by historical ANCA status, baseline BVAS, and baseline VDI score

**Figure 1:**
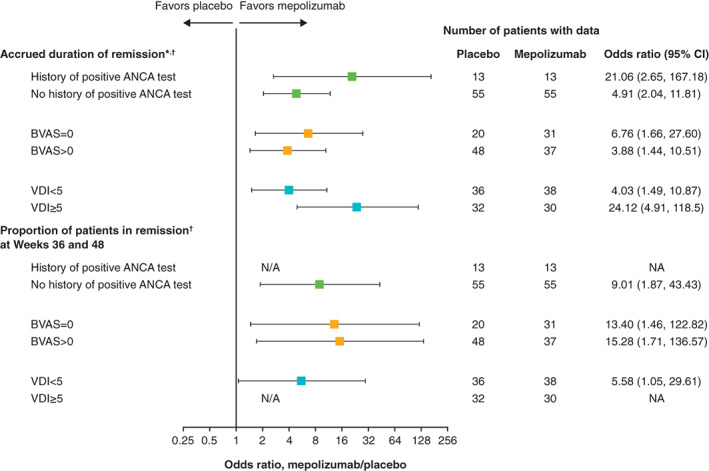
Accrued duration of remission and proportion of patients in remission at Weeks 36 and 48 by historical ANCA status, baseline BVAS, and baseline VDI score. *Accrued number of weeks of remission over the 52‐week study period categorized in weeks (0, >0 to <12, 12 to <24, 24 to <36, and ≥36 weeks). ^†^Remission was defined as BVAS equal to 0 and OG dose 4 mg/day or more of a prednisolone equivalent. ANCA, antineutrophil cytoplasmic antibody; BVAS, Birmingham Vasculitis Activity Score; CI, confidence interval; NA, not available—estimate could not be calculated because no patients in the placebo group achieved remission at Weeks 36 and 48; VDI, Vasculitis Damage Index.

Of the 136 patients in the overall study population, 26 (19%) had a history of a positive ANCA test, 85 (63%) had a baseline BVAS of more than 0, and 62 (46%) had a baseline VDI score of 5 or greater, which were used as markers of vasculitic EGPA. Irrespective of history of ANCA positive status, baseline BVAS (=0 or >0), or baseline VDI score (<5 or ≥5), patients treated with mepolizumab accrued more time in remission versus those who received placebo (Figure 1; Table 2).

**Table 2:** Analysis of remission in patients stratified by historical ANCA status, baseline BVAS, and baseline VDI score

	History of positive ANCA test				Baseline BVAS				Baseline VDI score			
	Yes		No		BVAS = 0		BVAS > 0		VDI < 5		VDI ≥ 5	
	PBO	Mepo	PBO	Mepo	PBO	Mepo	PBO	Mepo	PBO	Mepo	PBO	Mepo
	(n = 13)	(n = 13)	(n = 55)	(n = 55)	(n = 20)	(n = 31)	(n = 48)	(n = 37)	(n = 36)	(n = 38)	(n = 32)	(n = 30)
Accrued duration of remission^a^ ^,^ ^b^ (wk), n (%)												
0	10 (77)	4 (31)	45 (82)	28 (51)	16 (80)	10 (32)	39 (81)	22 (59)	27 (75)	17 (45)	28 (88)	15 (50)
>0 to <12	2 (15)	0	6 (11)	8 (15)	2 (10)	3 (10)	6 (13)	5 (14)	5 (14)	6 (16)	3 (9)	2 (7)
12 to <24	0	4 (31)	3 (5)	5 (9)	0	8 (26)	3 (6)	1 (3)	2 (6)	6 (16)	1 (3)	3 (10)
24 to <36	0	2 (15)	0	8 (15)	0	3 (10)	0	7 (19)	0	4 (11)	0	6 (20)
≥36	1 (8)	3 (23)	1 (2)	6 (11)	2 (10)	7 (23)	0	2 (5)	2 (6)	5 (13)	0	4 (13)
OR (95% CI)^c^	21.06 (2.65, 167.18)		4.91 (2.04, 11.81)		6.76 (1.66, 27.60)		3.88 (1.44, 10.51)		4.03 (1.49, 10.87)		24.12 (4.91, 118.50)	
Proportion of patients in remission^b^ at Weeks 36 and 48, n (%)	0	7 (54)	2 (4)	15 (27)	1 (5)	14 (45)	1 (2)	8 (22)	2 (6)	11 (29)	0	11 (37)
OR (95% CI)^c^	NA		9.01 (1.87, 43.43)		13.40 (1.46, 122.82)		15.28 (1.71, 136.57)		5.58 (1.05, 29.61)		NA	

Abbreviations: ANCA, antineutrophil cytoplasmic antibody; BVAS, Birmingham Vasculitis Activity Score; CI, confidence interval; Mepo, mepolizumab; NA, not applicable—estimate could not be calculated owing to lack of patients in the placebo group achieving remission at Weeks 36 and 48; OR, odds ratio; PBO, placebo; VDI, Vasculitis Damage Index.

^a^ Accrued number of weeks of remission over the 52‐week study period categorized in weeks (0, >0 to <12, 12 to <24, 24 to <36, and ≥36 weeks).

^b^ Remission was defined as BVAS = 0 and oral glucocorticoid dose ≤4 mg/day of a prednisone equivalent.

^c^ Mepolizumab versus placebo.

Across all ANCA, BVAS, and VDI subgroups, a larger proportion of patients were in remission at both Weeks 36 and 48 with mepolizumab versus placebo (Figure 1; Table 2). These proportions (mepolizumab vs. placebo) were as follows: 54% (n = 7) versus 0% for patients with a history of ANCA positivity and 27% (n = 15) versus 4% (n = 2) for patients without a history of ANCA positivity; 45% (n = 14) versus 5% (n = 1) for patients with a BVAS equal to 0 and 22% (n = 8) versus 2% (n = 1) for patients with a BVAS of more than 0; and 29% (n = 11) versus 6% (n = 2) for patients with a VDI score of less than 5 and 37% (n = 11) versus 0% for patients with a VDI score of 5 or greater. Patients with ANCA positivity, a BVAS of more than 0, and a VDI score of 5 or greater are more likely to have a vasculitic EGPA phenotype.

### Interaction between mepolizumab treatment effect and baseline variables

Baseline BVAS in the study population ranged from 0 to 22 with a median of 1.0, and the mean was 3.3 with a standard deviation (SD) of 4.70. There was no evidence that the mepolizumab treatment effect on accrued duration of remission differed by baseline BVAS when assessed as a continuous (*P* = 0.809) or categorical (defined as BVAS = 0 vs. BVAS > 0; *P* = 0.458) variable. Similar results were obtained for the proportion of patients in remission at both Weeks 36 and 48 (BVAS as a continuous variable: *P* = 0.736; BVAS as a categorical variable: *P* = 0.982).

Baseline VDI score in the study population ranged from 0 to 21 with a median of 4.0, and the mean was 4.6 with a SD of 3.10. There was no evidence that the mepolizumab treatment effect on accrued duration of remission differed by baseline VDI score, when assessed as a categorical variable (defined as VDI score <5 vs VDI score ≥5; *P* = 0.170). There was some evidence that the effect of mepolizumab treatment on the proportion of patients in remission at both Weeks 36 and 48 differed by baseline VDI score (*P* = 0.058); however, the treatment effect could not be estimated in patients with a baseline VDI score of 5 or greater because there were no patients in remission at Weeks 36 and 48 receiving a placebo within this subgroup.

### Effect of mepolizumab on EGPA relapse, and by type of relapse

**Figure 2:**
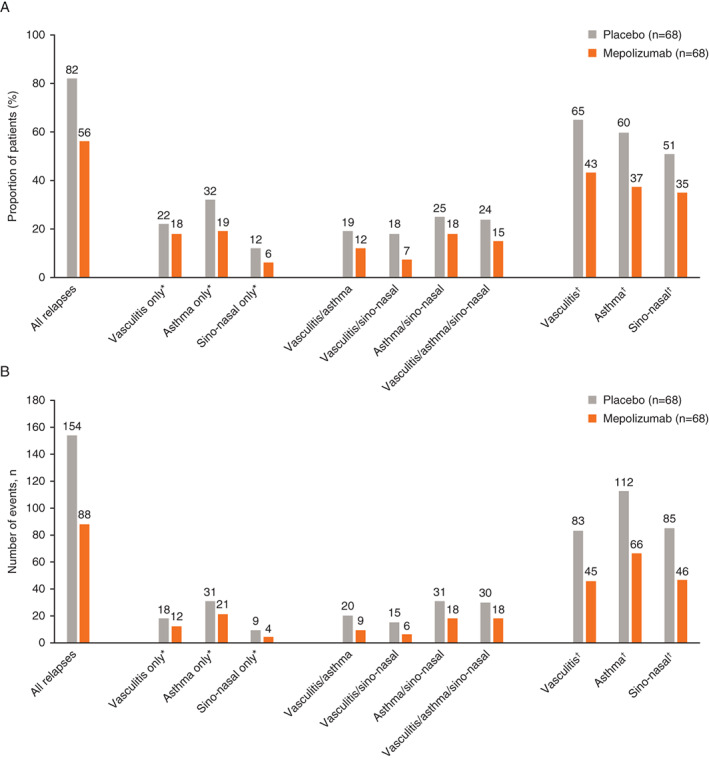
Proportion of patients experiencing a relapse (**A**) and number of relapse events (**B**) by type of relapse. *Relapse category selected without another relapse category defined. ^†^Relapse category selected with or without another relapse category defined; vasculitis relapse defined as BVAS of more than 0; asthma relapse defined as active asthma symptoms and/or signs with a corresponding worsening in ACQ‐6 score (compared with the most recent previous score); sino‐nasal relapse defined as active nasal and/or sinus disease, with corresponding worsening in one or more of the sino‐nasal symptom questions (compared with the most recent previous assessment). ACQ‐6, Asthma Control Questionnaire 6; BVAS, Birmingham Vasculitis Activity Score.

**Figure 3:**
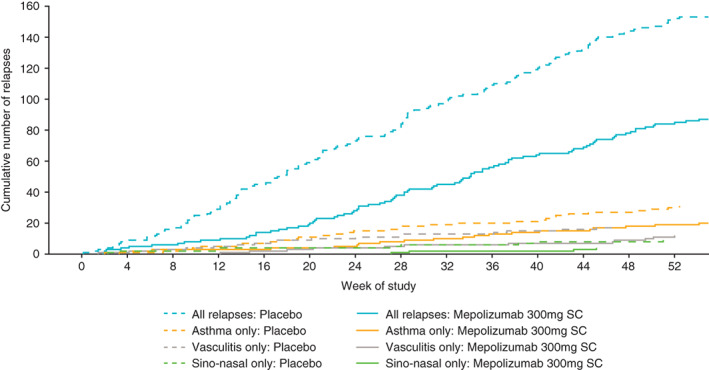
Cumulative number of EGPA relapses and by relapse type. For patients who withdrew from treatment, observed data following treatment discontinuation were used where available. Relapse categories are selected without another relapse category defined; asthma relapse defined as active asthma symptoms and/or signs with a corresponding worsening in ACQ‐6 score (compared with the most recent previous score); vasculitis relapse defined as BVAS of more than 0; sino‐nasal relapse defined as active nasal and/or sinus disease, with corresponding worsening in one or more of the sino‐nasal symptom questions (compared with the most recent previous assessment). ACQ‐6, Asthma Control Questionnaire 6; BVAS, Birmingham Vasculitis Activity Score; EGPA, eosinophilic granulomatosis with polyangiitis; SC, subcutaneous.

A lower proportion of patients experienced EGPA relapses with mepolizumab versus placebo; these relapses included vasculitic (BVAS > 0) only (no active asthma symptoms, no worsening in ACQ‐6 or sino‐nasal symptom questions scores [18% vs. 22%]) and vasculitis with or without another relapse category defined (43% vs. 65%), respectively. Similarly, patients receiving mepolizumab versus placebo also experienced a lower number of relapse events (Figure 2). Patients in the mepolizumab group had a lower cumulative number of all types of EGPA relapses than patients in the placebo group; differences between the mepolizumab and placebo groups according to individual relapse type (asthma only, vasculitis only, sino‐nasal only) were markedly smaller (Figure 3).

Patients receiving mepolizumab had accrued more time during which their BVAS was equal to 0 (odds ratio [95% confidence interval]: 3.71 [1.82‐7.60]; *P* < 0.001). In total, 50% of patients had a BVAS equal to 0 at Weeks 36 and 48 in the mepolizumab group versus 34% in the placebo group (*P* = 0.092; Table 3).

**Table 3:** Accrued duration of BVAS = 0 and proportion of patients achieving BVAS = 0 during the treatment period

	PBO	Mepo
	(n = 68)	(n = 68)
Accrued duration of BVAS = 0 over 52 wk, n (%)		
0	6 (9)	3 (4)
>0 to <12 wk	15 (22)	13 (19)
12 to <24 wk	11 (16)	5 (7)
24 to <36 wk	17 (25)	2 (3)
≥36 wk	19 (28)	45 (66)
OR (95% CI)^a^	3.71 (1.82, 7.60);	
*P* value	*P* < 0.001	
Proportion of patients with BVAS = 0 at Weeks 36 and 48, n (%)	23 (34)	34 (50)
OR (95% CI)^a^	1.94 (0.90, 4.19);	
*P* value	*P* = 0.092	

Abbreviations: BVAS, Birmingham Vasculitis Activity Score; CI, confidence interval; Mepo, mepolizumab; OR, odds ratio; PBO, placebo.

^a^ Mepolizumab versus placebo.

## DISCUSSION

Using data from the MIRRA study, this is the first analysis to evaluate the clinical benefit of mepolizumab in patients with a predominantly vasculitic EGPA phenotype (29). Irrespective of historical ANCA positive status, baseline BVAS, or baseline VDI score, patients treated with mepolizumab demonstrated a greater accrued duration of remission, and more patients treated with mepolizumab were in remission at both Weeks 36 and 48 than those who received placebo. Overall, the presence and/or history of vasculitic characteristics were generally similar in patients achieving remission and in those who did not achieve remission. Patients treated with mepolizumab also had fewer EGPA relapses including vasculitis events and more weeks without vasculitic disease activity. Together, these results suggest that both patients with and those without a vasculitic EGPA phenotype achieve clinical benefits with mepolizumab.

The results of this analysis expand on those previously demonstrated in the primary analysis of the MIRRA study, which demonstrated that patients with EGPA treated with mepolizumab had a greater accrued duration of remission versus placebo, with one‐third of mepolizumab‐treated patients in remission at both Weeks 36 and 48 (29). Additionally, increased duration of remission with mepolizumab over placebo has been demonstrated using alternative definitions of remission (30). Furthermore, in a subgroup of patients with EGPA and blood eosinophil counts of less than 150 cells/μL, mepolizumab increased the duration of remission (29, 30) although treatment benefits were more limited than in patients with counts of 150 cells/μL or more, potentially due to higher baseline OG doses in the former versus latter group (25, 28). In the current analysis, remission benefits with mepolizumab over placebo were observed in patients with and without features of a vasculitic phenotype including those with and without a history of ANCA positivity. This suggests that, although ANCA is often associated with the vasculitic EGPA phenotype (5, 6) and may directly contribute to disease pathology (6, 33), mepolizumab can facilitate remission via targeting eosinophils and their contribution to disease activity (4, 6). Accordingly, ANCA binding level does not always correlate with disease severity, ANCA can persist in disease remission, and some patients with vasculitic manifestations may test ANCA negative (34, 35, 36). In addition to the results by ANCA status, both patients with baseline BVAS greater than 0 and those with VDI scores of 5 or greater (features of vasculitic activity) also achieved a longer duration of accrued remission, and more of these patients were in remission at both Weeks 36 and 48 with mepolizumab compared with placebo. It should be noted that some vasculitic components were based on historical reports and may not represent active disease at the time mepolizumab was started. Furthermore, patients with organ‐ or life‐threatening manifestations in the 3 months before screening were not included in MIRRA. Nevertheless, taken together, these data support the use of mepolizumab in patients with EGPA with and without a vasculitic phenotype.

The proportion of patients with EGPA vasculitic disease components including neuropathy (39% to 43%), ANCA positivity (16% to 24%), palpable purpura (12% to 13%), alveolar hemorrhage (2% to 4%), and glomerulonephritis (0% to 1%) did not differ with remission status. Consistent with this, the proportion of patients who accrued 36 weeks of remission or longer was generally similar. Taken together these data suggest that the presence of the examined EGPA vasculitic components may not predict patient remission outcomes to mepolizumab. Additionally, the finding regarding ANCA status broadly supports the European EGPA study group consensus statement, which suggests that ANCA status by itself should not guide treatment decisions (34). The results are also informative for physicians when guiding treatment selection for remission induction in patients with active, nonsevere disease (ie, patients without life‐ or organ‐threatening manifestations, an area the American College of Rheumatology/Vasculitis Foundation guideline for ANCA‐associated vasculitis has outlined as requiring further investigation) (10).

Mepolizumab reduced the proportion of patients with all types of EGPA relapses, including vasculitis, asthma, and sino‐nasal relapses, compared with placebo, in addition to the cumulative number of all types of relapses. The events categorized into vasculitic only had concurrent asthma and sino‐nasal relapses removed; therefore, any influence by these events was likely excluded. Relapses are events that some patients continue to experience despite treatment with systemic glucocorticoids, immunosuppressants, or cytotoxic therapy (12, 14, 15, 18). Additionally, patients treated with mepolizumab also achieved a greater total accrued number of weeks with a BVAS of 0, indicating no vasculitic disease activity. This is consistent with previously reported results for patients with EGPA in Japan, including data reported for an interim analysis of a post‐marketing surveillance study, and these studies showed improvements in vasculitic symptoms based on BVAS following mepolizumab treatment (37, 38). These results are also in accordance with EGPA treatment goals to increase remission and reduce relapse rates, in addition to minimizing OG exposure (2). Another focus is on reducing organ damage, which can accumulate even several years after the EGPA diagnosis owing to continued disease activity and prolonged OG use (39). Organ damage can also lead to persistent symptoms, and patients can potentially become less responsive to further treatment (39).

This analysis has several limitations, which should be considered when interpreting results. Firstly, although a biopsy is recommended to support a diagnosis of vasculitis (9), biopsy criteria could not be used in the current analysis to support the identification of a vasculitic phenotype. This was because the historical biopsy eligibility criteria used in MIRRA included features that were not specific to vasculitic EGPA, including evidence of eosinophilic vasculitis, perivascular eosinophilic infiltration, and eosinophil‐rich granulomatous inflammation. Secondly, the most common criteria suggestive of vasculitic disease was neuropathy (41% of all MIRRA patients; mononeuropathy or polyneuropathy) (29). However, as neuropathy can be caused by either vasculitis or eosinophilic tissue infiltration (6), the possibility that patients had neuropathy of eosinophilic origin cannot be fully excluded. Thirdly, the BVAS tool has limitations, including a lack of specificity for vasculitis because asthma components such as wheezing could also be captured within the tool, which means that patients could have had a BVAS of greater than than 0 due to their asthma rather than vasculitis symptoms. BVAS also captures features that are more often attributed to tissue eosinophilia than vasculitis, such as cardiomyopathy (18, 40). Additionally, BVAS data were collected as total scores and not by individual components, and it was not possible to remove nonvasculitis components from BVAS data due to the lack of adjudication. Fourthly, although no evidence of interaction was seen between the baseline BVAS and the accrued duration of remission or proportion of patients in remission at both Weeks 36 and 48, there was some evidence that the mepolizumab treatment effect on the latter endpoint differed by baseline VDI score. However, given that no patients who were receiving placebo in the subgroup with a VDI score of 5 or greater achieved remission at both Weeks 36 and 48, results for this endpoint and subgroup should be interpreted with caution. Another limitation is that vasculitis features are most commonly present at the time of EGPA diagnosis, but the MIRRA eligibility criteria excluded any new EGPA diagnoses, which may have reduced the proportion of patients with vasculitis features. Finally, the study design encouraged recruitment of patients with established disease, particularly those with asthma and sino‐nasal disease, leading to small sample sizes for some analyses, including in the placebo group.

In this post hoc analysis of data from the MIRRA study, patients with EGPA and a predominantly vasculitic phenotype had similar mepolizumab treatment outcomes as those without a vasculitic phenotype. Moreover, patients achieving and not achieving remission were similar in terms of their history or presence of EGPA diagnostic features. These results suggest patients with EGPA and a vasculitic phenotype are likely to achieve clinical benefits with mepolizumab, thus providing valuable information for clinicians treating patients with EGPA with and without vasculitic phenotypes.

## AUTHOR CONTRIBUTIONS

All authors were involved in drafting the article or revising it critically for important intellectual content, and all authors approved the final version to be published. All authors had full access to all of the data in the study and take responsibility for the integrity of the data and the accuracy of the data analysis.

### Acquisition of data

Terrier, Jayne, Hellmich, Akuthota, Khoury, Weschler.

### Study conception and design

Steinfeld, Yancey.

### Analysis and interpretation of data

Terrier, Jayne, Hellmich, Bentley, Steinfeld, Yancey, Kwon, Akuthota, Khoury, Baylis, Weschler.

## Supporting information


Disclosure form

**Appendix S1:** Supplementary Information
