# Supplementary material for: Clinical Benefit of Mepolizumab in Eosinophilic Granulomatosis With Polyangiitis for Patients With and Without a Vasculitic Phenotype
Source: ACR Open Rheumatol. 2023 Jun 13;5(7):354–63. doi: 10.1002/acr2.11571 (PMC10349249; doi:10.1002/acr2.11571)
Supplement: Supplementary file 2 — Appendix S1: Supplementary Information [file ACR2-5-354-s002.docx]

**Supplementary material**

**EGPA mepolizumab study team:**

- Chiara Baldini, Centro Farmacologia Clinica AOUP, Pisa, Italy; Douglas M Beach, Beth Israel Deaconess Medical Center, Boston, MA, USA;
- Bruce Bochner, Northwestern University; Arnaud Bourdin, Hôpital Arnaud de Villeneuve, Montpellier, France;
- Pascal Chanez, Hôpital Nord, Marseille, France; Anoop Chauhan, Queen Alexandra Hospital, Portsmouth, UK; Vincent Cottin, Hôpital Louis Pradel, Bron, France;
- Maria C Cid, the Vasculitis Research Unit, Department of Autoimmune Diseases, Hospital Clinic University of Barcelona, Institut d’Investigacions Biomèdiques August Pi i Sunyer, Barcelona, Spain;
- Bertrand Dunogue, Hôpital Cochin, Paris, France; Georgina Espígol-Frigolé, Department of Autoimmune Diseases, Hospital Clínic University of Barcelona, Institut d’Investigacions Biomèdiques August Pi I Sunyer (IDIBAPS), Barcelona, Spain;
- Aryeh Fischer, National Jewish Health, Denver, CO, USA;
- Gerald J Gleich, the Departments of Dermatology and Medicine, University of Utah School of Medicine, Salt Lake City, UT, USA;
- Rula Hajj-Ali, Cleveland Clinic, Cleveland, OH, USA; Berhnard Hellmich, Kreiskliniken Esslingen Klinik Kirchheim, Kirchheim unter Teck, Germany;
- José Hernández Rodríguez, Department of Autoimmune Diseases, Hospital Clínic University of Barcelona, Institut d’Investigacions Biomèdiques August Pi I Sunyer (IDIBAPS), Barcelona, Spain;
- Julia Holle, Rheumazentrum Schleswig-Holstein Mitte, Neumünster Germany;
- Christof Iking-Konert, Rheumaklinik Bad Bramstedt & Universitaetsklinikum Schleswig-Holstein, Bad Bramstedt, Germany; Tomonori Ishii, Tohoku University Hospital, Miyagi, Japan;
- Jean-Emmanuel Kahn, Hôpital Foch, Suresnes, France; Peter Kern, Klinikum Fulda-MVZ Osthessen, Hessen, Germany; Nader Khalidi, St. Joseph's Healthcare Hamilton, Hamilton, ON, Canada;
- Amy Klion, the Human Eosinophil Section, Laboratory of Parasitic Diseases, National Institute of Allergy and Infectious Diseases, National Institutes of Health, Bethesda, MD;
- Curry L Koening, Department of Medicine, University of Utah, School of Medicine, Salt Lake City, UT, USA;
- Carol A Langford, the Center for Vasculitis Care and Research, Cleveland Clinic, Cleveland, USA;
- Andrea Matucci, Azienda Ospedaliero-Universitaria di Careggi, Firenze, Italy;
- Peter A. Merkel, the Division of Rheumatology and the Department of Biostatistics and Clinical Epidemiology, University of Pennsylvania; USA;
- Frank Moosig; Rheumazentrum, Schleswig–Holstein Mitte, Neumünster, Germany;
- Paul Monach, Boston University School of Medicine, Boston, MA, USA;
- Thomas Neumann, Klinikum der Friedrich Schiller Universitaet Jena, Thueringen, Germany;
- Christian Pagnoux, Mount Sinai Hospital, Toronto, ON, Canada;
- Elide Pastorello, Azienda Ospedaliera Ospedale Niguarda Cà Granda, Milano, Italy;
- Sergio Prieto-González, Department of Autoimmune Diseases, Hospital Clínic University of Barcelona, Institut d’Investigacions Biomèdiques August Pi I Sunyer (IDIBAPS), Barcelona, Spain;
- Xavier Puechal, Hôpital Cochin, Paris, France; Benjamin A. Raby, Brigham & Women’s Hospital, Boston, MA, USA;
- Florence Roufosse, Hôpital Erasme, Bruxelles, Belgium; Maria Grazia Sabbadini, Ospedale San Raffaele IRCCS, Milano, Italy;
- Jan Schirmer, University Hospital of Kiel, Kiel, Germany;
- Ulrich Specks; the Division of Pulmonary and Critical Care Medicine, Mayo Clinic, Rochester, MN, USA;
- Antoine G Sreih, University of Pennsylvania, Philadelphia, PA, USA;
- Masami Taniguchi, Sagamihara National Hospital, Kanagawa, Japan; Reinhard Voll, Universitaetsklinikum Freiburg, Freiburg, Germany;
- Andrew Wardlaw, Respiratory Biomedical Research Unit, Glenfield Hospital, Leicester, UK;
- Peter F Weller, Beth Israel Deaconess Medical Center, Boston, MA, USA.
